# Comparative performance of FINDRISC and IDRS in screening for type 2 diabetes risk among Yemeni university students

**DOI:** 10.1186/s13098-026-02123-1

**Published:** 2026-04-02

**Authors:** Abdulsalam Al-Shami, Lina Qasem, Mufeed Baddah, Sami Kahtan

**Affiliations:** 1 Department of Clinical Biochemistry, Faculty of Medicine and Health Sciences, Taiz University, Taiz, Yemen; 2 Department of Medical Biochemistry, Faculty of Medicine and Health Sciences, Amran University, Amran, Yemen; 3 Department of Clinical Biochemistry, Faculty of Medicine and Health Sciences, University of Aden, Aden, Yemen

**Keywords:** Type 2 diabetes mellitus, FINDRISC, IDRS, Dysglycemia, Yemen, Diagnostic accuracy

## Abstract

**Background:**

Type 2 diabetes mellitus (T2DM) is a leading cause of death worldwide.To address the rise of T2DM, it might be more effective to create and validate a targeted risk scoring system for specific populations. This study aimed to evaluate and compare the diagnostic accuracy of the Finnish Diabetes Risk Score (FINDRISC) and the Indian Diabetes Risk Score (IDRS) in screening for dysglycemia (i.e. the presence of prediabetes or T2DM) and to identify the factors associated with T2DM risk among young medical students at the Faculty of Medicine, Taiz University, Yemen.

**Methods:**

A cross-sectional study was conducted among 200 medical students without a prior history of diabetes at Taiz University. The FINDRISC and IDRS questionnaires were used along with fasting blood sugar to estimate the efficacy of both risk scores in screening dysglycemia. Descriptive statistics and the chi-square test were used, with *P* < 0.05 regarded as statistically significant. The diagnostic accuracy of FINDRISC and IDRS was compared using the area under the receiver operating characteristic curve (AUC-ROC). Sensitivity, specificity, and the best cut-off to detect dysglycemia were calculated for each risk score. The best cutoffs were determined by maximizing the Youden Index.

**Results:**

of the 200 participants, 10.5% and 1.5% were diagnosed with prediabetes and T2DM, respectively, where females had a higher prevalence than males for both outcomes (*P* < 0.001). The screening performance for dysglycemia differed significantly between the two tools (*P* < 0.001). The FINDRISC achieved a larger AUC-ROC (0.782; 95% CI: 0.68–0.88) with **≥** 9 as an optimal cut-off (sensitivity: 67.0%, specificity: 80.1%, and Youden index: 0.44) compared to that of IDRS (0.671; 95% CI: 0.56–0.78) with **≥** 45 as an optimal cut-off (sensitivity: 46.0%, specificity: 80.0%, Youden Index: 0.25). A Bland-Altman plot indicated adequate agreement at lower risk scores; however, significant divergence at higher risk scores, suggesting the tools are not interchangeable.

**Conclusion:**

The FINDRISC demonstrated acceptable diagnostic utility and significantly outperformed the IDRS in this young Yemeni population;with its effectiveness increasing at higher risk scores. This makes it the preferred initial screening tool for identifying individuals with dysglycemia, especially in resource-constrained environments, such as the Yemeni setting. However, due to its modest standalone sensitivity, we recommend that individuals with a FINDRISC score ≥ 9 should undergo definitive laboratory testing to confirm the diagnosis and ensure early detection of dysglycemia.

## Background

Diabetes mellitus (DM) is a group of metabolic disorders characterized by high blood glucose levels due to impairments in insulin production (type 1 diabetes), or insulin action (type2 diabetes) [1, 2]. Type 2 Diabetes Mellitus (T2DM) is the most prevalent type of diabetes, which is responsible for almost 90% of the total cases of diabetes worldwide [3].

Diabetes has been increasingly identified as a serious, global public health challenge [4]. According to the Diabetes Atlas (2022) published by the International Diabetes Federation (IDF), the number of individuals with diabetes mellitusworldwide currently around 537 million and is projected to grow to 783 million by 2045, with the highest proportion observed in low- and middle-income countries [4]. The increasing worldwide prevalence of DM represents substantial economic burdens. Globally, health care costs estimate in 2021 were 966 billion USD, and are estimated to be increased to 1,054 billion USD by 2045 [4]. Unfortunately, besides a rapidly increased incidence among young individuals, more than 50% of the diabetics in the world remain unaware of their illness status, which contributes to the disease burden by increasing a public health risk and preventing immediate interventions [4, 5].

In Yemen, our knowledge about the prevalence of T2DM remains poor with little data available. Although; it has been found that the prevalence of T2DM in Yemen was 4.6% by 2004, the number increased to 10.4% in 2008 [6, 7]. The rise in T2DM in Yemen can be attributed to the genetic predisposition, urbanization, the sedentary life-styles and the changing food habits. Given that diabetes is a chronic disease associated with a high rate of mortality, and health care expenditures that have been explained by long-term vascular complications, thus managing T2DM represents one of the biggest worldwide health concerns at the present time [8, 9]. Hence, an urgent need has emerged to develop a simple, fast, cost-effective and non-invasive screening tool for early identification of individuals at higher risk of developing T2DM in the future [10, 11].

Previous studies demonstrated that early screening and detection, diagnosis and management of the risk of T2DM could alleviate the rapidly growing socioeconomic burdens of T2DM, thus delaying or preventing the development of the illness and reducing serious complications [12–14]. Considering the targeted interventions such as lifestyle modification and exercise or medications, lifestyle modifications are proven to be beneficial to avoid T2DM and lessen its burden, thus improving health care outcomes and the quality of life [15].

Recently, various diabetic risk scores have been developed to identify individuals with undiagnosed T2DM (prevalent), or those who are at risk of developing T2DM (incident) [16]. Some diabetic assessment models have been validated in selected populations, prompting their use in other countries. However, recent studies have shown that these risk scoring systems derived from the same populations may not be appropriate for other ethnic groups [17]; therefore, there is a need to establish a diabetes risk score for the Yemeni population.

To the best of our knowledge, no previous studies have been conducted to compare two different existing diabetes risk screening tools in the adult Yemeni population during wartime. Hence, the current study was designed to evaluate and compare the diagnostic accuracy of the Finnish Diabetes Risk Score (FINDRISC) and the Indian Diabetes Risk Score (IDRS) in screening for dysglycemia and undiagnosed T2DM (UT2DM) among healthy medical students at the Faculty of Medicine and Health Sciences, Taiz University, Yemen. We also determine the factors associated with the risk of T2DM among study subjects.

## Methods

### Study design and setting

A cross-sectional study was conducted among 200 medical students during a three-month period (January to March 2024) at the Faculty of Medicine and Health Sciences, Taiz University, Yemen. Taiz is the third largest city in the country and is located in the central of its southwestern highlands region, 1400 m (4,600 ft) above sea level.

### Study population

During the study period, second and third-year medical students were recruited using a convenience sampling technique at the Faculty of Medicine and Health Sciences, Taiz University. Students with previously diagnosed T2DM, incomplete questionnaires, refusal to participate, or absence during data collection were excluded.

### Sample size

The sample size was calculated using the following formula:

Where N = Sample size, a 95% confidence level (Z = 1.96), a percentage of failure (q = 1 – p), and an estimated prevalence (P) of 50% was used due to lacking of available data on the prevalence of prediabetes mellitus (PDM) among Yemeni medical students with a 5% margin of sampling error (d = 5%). The formula was applied, resulting in N = (1.96)^2^ × 0.5 × 0.5/(0.05)^2^ = 384. To account for the finite population size, the Adjusted Sample Size formula was then applied. The population size (S) was set to 346, representing the total number of second and third-year medical students during the study period. The adjusted sample size was calculated as N = [(384)/(1 + (384–1)/(346)] = 181. Considering a 5% non-response rate, the minimum recommended sample size for the study was determined to be 190.

### Study tool

There has not been a previously validated risk scoring system for the Yemeni population. After a review of validated risk assessment scoring systems for different countries [16, 17]. The current study used two diabetes risk scores: Finnish Diabetes Risk Score (FINDRISC) and Indian Diabetes Risk Score (IDRS) for assessing the risk of developing T2DM within the next 10 years.

The Indian Diabetes Risk Score (IDRS) considers four risk factors; age, waist circumference (WC), physical activity and family history to assess the risk of developing T2DM [18]. Based on the total score, participants are classified into different risk levels: a score of < 30 indicates low risk, 30 to 59 denotes moderate risk, and a score exceeding 60 represents high risk of developing diabetes (Tables 2 and 3) [10, 19]. In the current study, the age component of the IDRS provided zero score for the entire population, as the age of all participants was under the age of 35. Consequently, the IDRS’s discriminatory power based solely on the remaining three components: WC, physical activity, and family history of diabetes.

The FINDRISC tool is a validated and frequently used questionnaire that consists of eight variables; age, BMI, WC, physical activity, daily consumption of fruit and vegetables, high blood pressure or antihypertensive medication, high fasting blood sugar level, and family history of diabetes [13, 20]. Participants with FINDRISC score of < 7 was categorized as low risk (estimated 1 in 100{1%} will develop T2DM), 7–11 as slightly elevated risk (estimated 1 in 25{4%} will develop T2DM), 12 to 14 as moderate risk (estimated 1 in 6{16%} will develop T2DM), 15 to 20 as high risk (estimated 1 in 3{33%} will develop T2DM) and those with a score > 20 as very high risk for developing diabetes (estimated 1 in 2{50%} will develop T2DM) (Table 4) [21]. Given that there is no a validated screening risk tool for Yemeni young population, we utilized both FINDRISC and IDRS cut-offs for BMI and WC. Previous studies provide scientific evidence that these cut-offs, which have been validated for European and South Asian populations, possess screening utility for T2DM in neighboring Arab populations. This justifies their use as suitable screening tools in the current study [22–24].

### Data collection

The participants data were obtained by using a predesigned, one page structured questionnaire containing participant’s name, age, gender, contact number, and different risk factors of T2DM that are essential to evaluate the discriminatory ability of the FINDRISC and IDRS tools.

After asking all participants to stand barefoot wearing light clothing, weight and height were measured in kilograms (to the nearest 0.1 kg) using a standard weighing scale, and in centimeters (to the nearest 0.5 cm) using a wall-mounted measuring tape respectively. BMI (kg/m^2^) was calculated by dividing weight (in kilograms) by height (in meters squared). Waist circumference was measured using a flexible measuring tape in centimeters (to nearest 0.1 cm) at halfway between the lowest rib and the superior border of the iliac crest.

Hypertension was defined asasys to lic blood pressure ≥140 mmHg and/or diastolic bloodpressure  ≥ 90 mmHg [25]. The blood pressure was measured by a manual sphygmomanometer in standard conditions (measured 2 times after a 5-min rest between each measurement [26].

### Blood glucose measurement

Participants were invited to the Laboratory of Clinical Biochemistry at the Faculty of Medicine and Health Sciences, Taiz University. Following an overnight fast (at least 8 h), fasting blood samples were initially collected via finger-pricking using a sterilized lancet; fasting blood sugar (FBS) levels were measured on the glucose test strip using a standard glucometer (Accu-Chek Active; Roche, Mannheim, Germany). To ensure diagnostic precision, results were validated using a semi-automatic biochemistry analyzer (BTS-330; BioSystems S.A., Barcelona, Spain) the enzymatic colorimetric glucose oxidase-peroxidase (GOD-POD) method on fasting venous samples. Based on FBS levels (mg/dl), participants were categorized as follows: normal (< 100 mg/dl) or dysglycaemia (prediabetes [PDM; 100–125 mg/dl], or diabetes mellitus [DM; ≥126 mg/dl]) [27].

### Data analysis

The collected data were entered into a computer database, and the statistical analysis was performed using SPSS version 26.0 software (SPSS Inc., Chicago, IL, USA). Descriptive statistics, including frequencies, proportions, means, and standard deviations, were used to summarize participant characteristics and the prevalence of diabetes risk factors. For categorical data, Pearson’s chi-square and the independent Student’s t-test were performed for continuous variables. Statistical significance was set at *P* **<** 0.05. To determine the optimal diagnostic cut-off values for the FINDRISC and IDRS in this population, the Youden Index (*J*) was applied. The optimal threshold was defined as the value that maximized the sum of sensitivity and specificity (*J* = Sensitivity + Specificity – 1). Diagnostic accuracy of both tools was also assessed using area under the ROC curve, sensitivity, specificity, and the Youden index. Additionally, positive likelihood ratio and negative likelihood ratio, as well as Mitchell’s clinical utility indices (CUIs) were calculated for each risk tool. Agreement between the two scoring systems in screening for dysglycemia and T2DM was analyzed by using the Bland–Altman plot (B-A plot).

## Results

### Characteristics of enrolled study subjects

A total of 200 participants were included, of whom110 (55.0%) were females and 90 (45.0%) were males. The mean (± SD) age of the entire population was 21.33 ± 1.75 years, with no statistically significant difference between females and males (21.3 ± 1.95 vs. 21.3 ± 1.48 years, respectively; *P* = 0.919). Of note, female participants had significantly higher mean values of body mass index (BMI; 22.6 ± 4.49 vs. 21.3 ± 3.43; *P* = 0.019), IDRS risk score (37.6 ± 17.51 vs. 24.2 ± 13.23; *P* < 0.001), and FINDRISC risk score (7.01 ± 3.53 vs. 4.1 ± 2.93; *P* < 0.001) compared to their male counterparts. Although females also showed a slightly higher mean values of fasting blood sugar (FBS; 85.1 ± 15.59 vs. 82.4 ± 13.66 mg/dl; *P* = 0.192) relative to males, the difference was not statistically significant.

On the other hand, the study results revealed that no significant differences were found between sexes regarding waist circumference (WC; females: 85.3 ± 8.94 vs. males: 86.0 ± 11.44; *P* = 0.588), systolic blood pressure (SBP; 110.3 ± 9.82 vs. 111.1 ± 10.64 mmHg; *P* = 0.583), or diastolic bloodpressure (DBP;77.0 ± 6.61 vs. 76.0 ± 7.57 mmHg; *P* = 0.326), as presented in Table 1.

**Table 1:** Baseline characteristics of study subjects*

Variable	Total (*n* = 200)	Females (*n* = 110)	Male (*n* = 90)	*P*-value
Age	21.3 ± 1.75	21.3 ± 1.95	21.3 ± 1.48	0.919
BMI (kg/m^2^)	22.0 ± 4. 10	22.7 ± 4. 50	21.3 ± 3. 42	0.019
WC (cm)	85.6 ± 10.11	85.3 ± 8.97	86.0 ± 11.39	0.588
FBS (mg/dl)	83. 9 ± 14.78	85.1 ± 15.59	82.4 ± 13.66	0.192
SBP (mmHg)	110.8 ± 10.26	111. 1 ± 10.64	110.3 ± 9.82	0.583
DBP (mmHg)	76. 5 ± 7.15	76.0 ± 7.57	77.0 ± 6.61	0.326
FINDRISC score	5.7 ± 3.61	7.0 ± 3.53	4.1 ± 2.93	< 0.001
IDRS score	32.1 ± 17.06	37.6 ± 17.51	24.2 ± 13.23	< 0.001

*Results are expressed as mean ± standard deviation. *p* < 0.05 considered as significant value

Age (years); BMI- Body mass index; WC -Waist circumstances; FBS -Fasting blood sugar; SBP- Systolic blood pressure; DBP- Diastolic Blood pressure; FINDRISC- Finnish Diabetes Risk Score; IDRS- Indian Diabetes Risk Score

### FINDRISC-Specific risk factors distribution

The distribution of FINDRIS diabetic risk factors among study subjects is summarized in Table 2. According to FINDRISC tool, 159(79.5%)had a healthyweight (BMI18.0–24.9 kg/m^2^), whereas 32 (16.0%) were overweight (BMI 25.0–29.9 kg/m²) and 9 (4.5%) were obese (BMI ≥ 30 kg/m²). Taken together, 41(20.5%) participants were either overweight or obese (BMI ≥ 25 kg/m^2^). The distribution of participants BMI by sex (Table 2) shows that 83 (75.5%) females and 76 (84.4%) males had a healthy weight, while 27(24.6%) females and 14 (15.5%) males were either overweight or obese. It is worth noting that the BMI difference observed between genders was statistically significant and higher in females than in males (*P* < 0.001).

**Table 2:** Distribution of FINDRISC- specific diabetic risk factors across gender among study participants^*^

Variable	FINDRISC Points	Total *n* = 200 (%)	Females *n* = 110 (%)	Male *n* = 90(%)	*p*-value	
Body Mass index (BMI; kg/m^2^)						
< 25	0	159 (79.5)	83 (75.5)	76 (84.4)		< 0.001
25–29.9.9	1	32 (16.0)	20 (18.2)	12 (13.3)		
≥ 30	3	9 (4.5)	7 (6.4)	2 (2.2)		
Consumption of vegetables or fruits						
Everyday	0	64 (32.3)	35 (31.8)	29 (32.2)		0.231
Not everyday	1	136 (68.0)	75 (68.2)	61 (67.8)		
Fasting blood Sugar						
< 100 mg/dl	0	176 (88.0)	94 (85.5)	82 (91.1)		< 0.001
100–125 or higher	5	24 (12.0)	16 (14.5)	8 (8.9)		

*Data are expressed as frequency and percentage *n* (%). Chi-Square *p* < 0.05 considered as significant value

None of the participants reported a history of anti- hypertensive medication anytime in their life. It was also observed that out of the 64 (32.3%) participants reported daily consumption vegetables or fruits, 35 (31.8%) and 29 (32.2%) were females and males, respectively, with no statistically significant difference between genders (*P* = 0.231; Table 2).

Regarding glycemic status, Table 2 shows that 176 (88.0%) participants had had normal fasting blood sugar (FBS) levels (< 100 mg/dl), while 24 (12.0%) reported elevated FBS levels (100–125 mg/dl or higher). Among these FBS levels (≥ 100 mg/dl), 16 (14.5%) were females and 8 (8.9%) were males, representing a statistically significant difference (*P* < 0.001). Overall, the prevalence of dysglycemia was 12.0%, with 24 participants in total. This case number exceeds the minimum requirement for robust AUC estimation, thereby providing sufficient precision for the primary diagnostic performance analysis.

### Common FIDRISC and IDRS risk factors distribution

The distribution of common FINDRISC and IDRS diabetic risk factors among study subjects are detailed in Table 3. Based on IDRS tool, all participants were young adults below the age of 35 years; thus, the age component contributed zero points to the risk score for this young population (*P* = 0.919).

In relation to WC distribution, out of 200 study subjects, 31 (28.2%) of females and 65 (72.2%) males had the low-risk thresholds of WC < 80 cm and < 94 cm, respectively. Of 38 (34.5%) females and 20 (22.2%) males who found to have the moderate-risk thresholds of WC (80–88 cm for females and 94–102 cm for males). Notably, 41 (37.3%) females and 5 (5.6%) males had the high-risk thresholds of WC > 88 cm and > 102 cm, respectively. Overall, 79 (39.5%) females and 25 (12.5%) males were categorized as having moderate-to-high risk thresholds of WC, indicating that central obesity was significantly more prevalent in females than in males (*P* < 0.001; Table 3). More than half of the participants (113; 56.5%) engaged in daily physical activity during either work or leisure time for a minimum of 30 min (*P* < 0.001). Unsurprisingly, a significantly higher proportion was observed in males (84.4% vs. 33.6%; *P* < 0.001) compared to their female counterparts.

Moreover, a high prevalence family history of diabetes was observed in this young population; overall, 84 (42.0%) reported having either first or second-degree relative, or both with diabetes. Among these, 73 (36.5%) had a positive family history in either a first-degree (parent, sister, brother, or child) or a second-degree relative (grandparent, uncle, aunt, or first cousin), while a dual family history (both parents or both first and second-degree) was reported by 5 (2.5%) and 6 (3.0%) the participants, respectively. Notably, 10.5% of participants had at least one parent with diabetes, while in 3.0% of participants, both parents had diabetes (*P* < 0.001; Table 3). Overall, a higher prevalence of positive family history was observed in females (43.6% vs. 40.0%) compared to their male counterparts.

**Table 3:** Distribution of common FINDRISC and IDRS diabetic risk factors across gender among study participants^*^

Variable	FINDRISC/IDRS Points	Total *n* = 200 (%)	Females *n* = 110 (%)	Male *n* = 90(%)	*p*-value	
**Waist circumference (cm; female/male)**						
< 80/94	0/0	96 (48.0)	31 (28.2)	65 (72.2)		< 0.001
80–88/94 − 102	2/10	58(29.0)	38 (34.5)	20 (22.2)		
> 88/102	3/20	46 (23.0)	41 (37.3)	5 (5.6)		
**Daily physical activity (30 min)**						
Regular/Vigorous	0/0–20^a^	113 (56.5)	37 (33.6)	76 (84.4)		< 0.001
No/Sedentary	2/30	87 (43.5)	73 (66.4)	14 (15.6)		
**Family History of Diabetes**						
No	0/0	116 (58.0)	62 (56.4)	54 (60.0)		< 0.001
Yes (Second degree only)	3/-	52 (26.0)	27 (24.5)	25 (27.8)		
Yes (First degree only/either parent)	5/10^b^	21 (10.5)	14 (12.7)	7 (7.8)		
Yes (First and second degree)^c^ Both Parents	5/-5/20^b^	5 (2.5) 6 (3.0)	4 (3.6) 3 (2.7)	1 (1.1) 3(3.3)		

* Data are presented as frequency and percentage *n* (%). Chi-Square *p* < 0.05 considered as significant value. ^a^ Based on IDRS criteria, physical activity is categorized as regular vigorous (score = 0), regular mild (score = 10), or sedentary (score = 20). ^b^ Positive family history in the IDRS is classified as either one parent (score = 10) or both parents (score = 20). ^C^ includes sister or brother

The screening performance for dysglycemia among the study subjects was assessed using the FINDRISC and IDRS scoring systems to categorize risk levels within the population (Table 4).

**Fig. 1:**
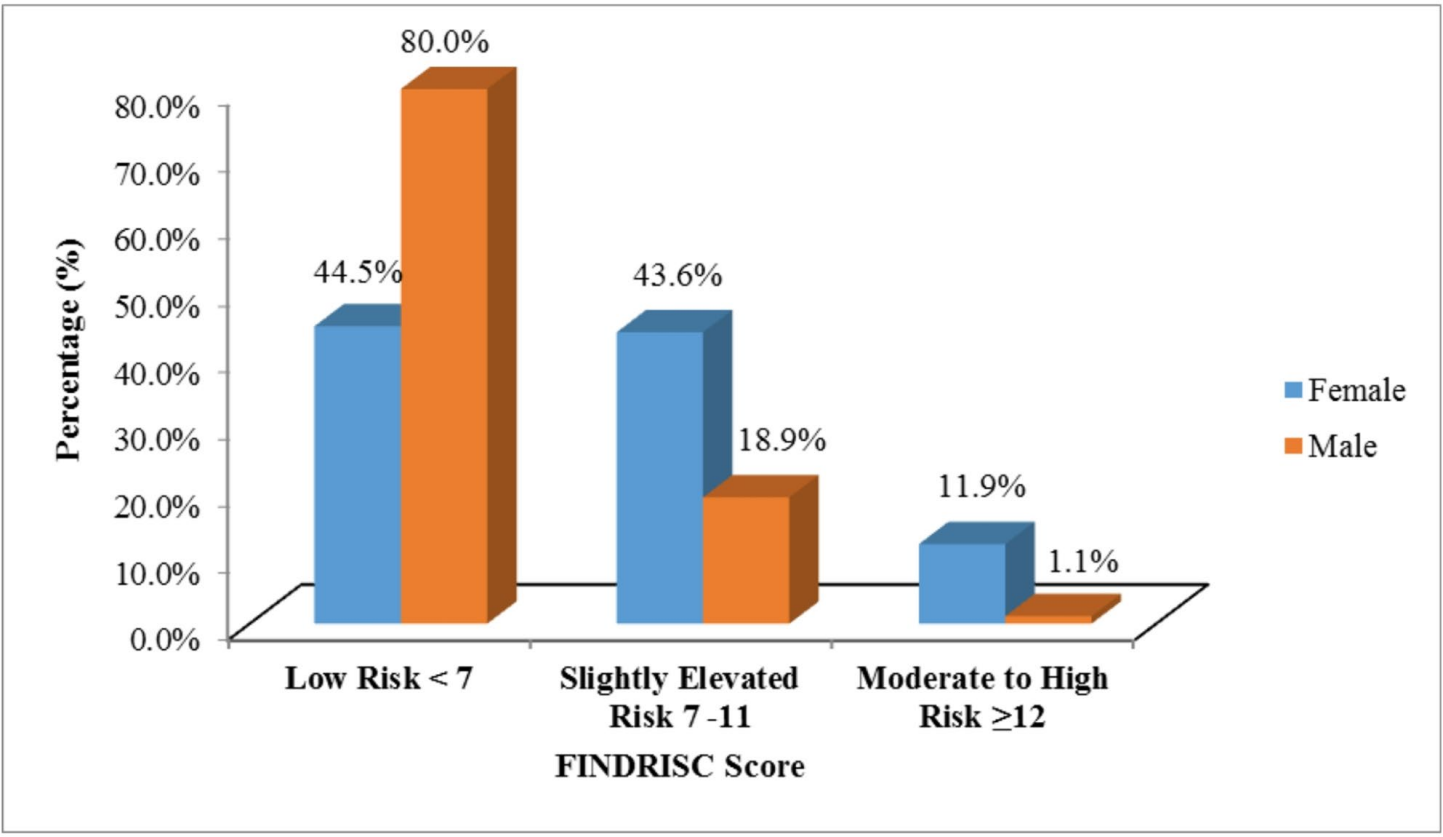
Distribution of dysglycemia risk based on total FINDRISC scores among medical students (*n* = 200)

Regarding the FINDRISC model (Table 4; Fig. 1), 121 (60.5%) students were at low risk (score < 7), 65 (32.5%) were at slightly elevated risk (score 7–11), 13 (6.5%) were at moderate (score 12–14) elevated risk, and 1 (0.5%) were at high risk (score 15–20). Taken together, out of the 14 (7.0%) participants found to have a moderate-to- high risk (score ≥ 12) for dysglycemia. The risk of dysglycemia was significantly higher among females (11.9% vs. 1.1%) than males, as indicated by a higher mean FINDRISC score (7.01 ± 3.53 vs. 4.10 ± 2.93; *P* < 0.001) (Table 1).

**Table 4:** Comparative distribution of risk factors and scoring components of FINDRISC and IDRS by gender

	Risk	Total *n* = 200 (%)	Females *n* = 110 (%)	Male *n* = 90(%)	*p*-value
**FINDRISC score**					
< 7	Low	121 (60.5)	49 (44.5)	72 (80.0)	< 0.001
7–11	Slightly elevated	65 (32.5)	48 (43.6)	17 (18.9)	
12–14	Moderate	13 (6.5)	12 (11.0)	1 (1.1)	
15–20	High	1 (0.5)	1.0 (0.9)	0 (0.0)	
**IDRS score**					
< 30	Low	83 (41.5)	29 (26.4)	54 (60.0)	< 0.001
30–59	Moderate	91 (45.5)	58 (52.7)	33 (36.7)	
≥ 60	High	26 (13.0)	23 (20.9)	3 (3.3)	

* Results are expressed as frequency and percentage *n* (%). Chi-Square, *p* < 0.05 considered as significant value

**Fig. 2:**
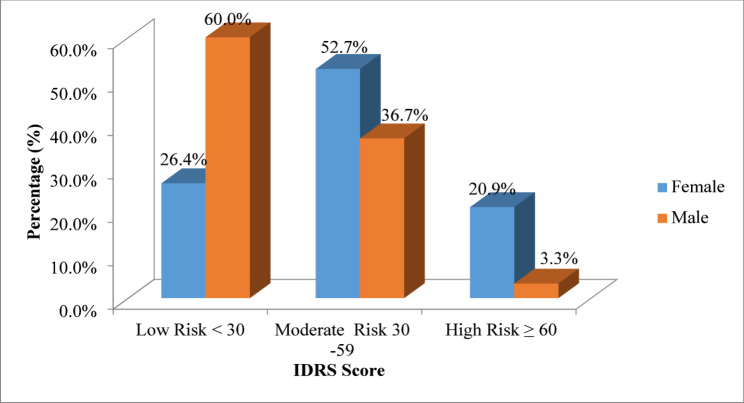
Distribution of dysglycemia risk based on total IDRS scores among medical students (*n* = 200)

According to the IDRS model, 83(41.5%) students were found to be at low risk (score < 30), 91(45.5%) were at moderate risk (score 30–59), and 26 (13.0%) were at high risk (score ≥ 60) as depicted in Table 4; Fig. 2. Collectively, a majority of the students (117; 58.5%) were classified as moderate to high risk (score ≥ 30) for dysglycemia. Similar to FINDRISC findings, female students had a significantly higher mean IDRS risk score compared to males (37.6 ± 17.51 vs. 24.2 ± 13.23; *P* < 0.001) (Table 1).

### Diagnostic accuracy of risk score models for undiagnosed T2DM

**Fig. 3:**
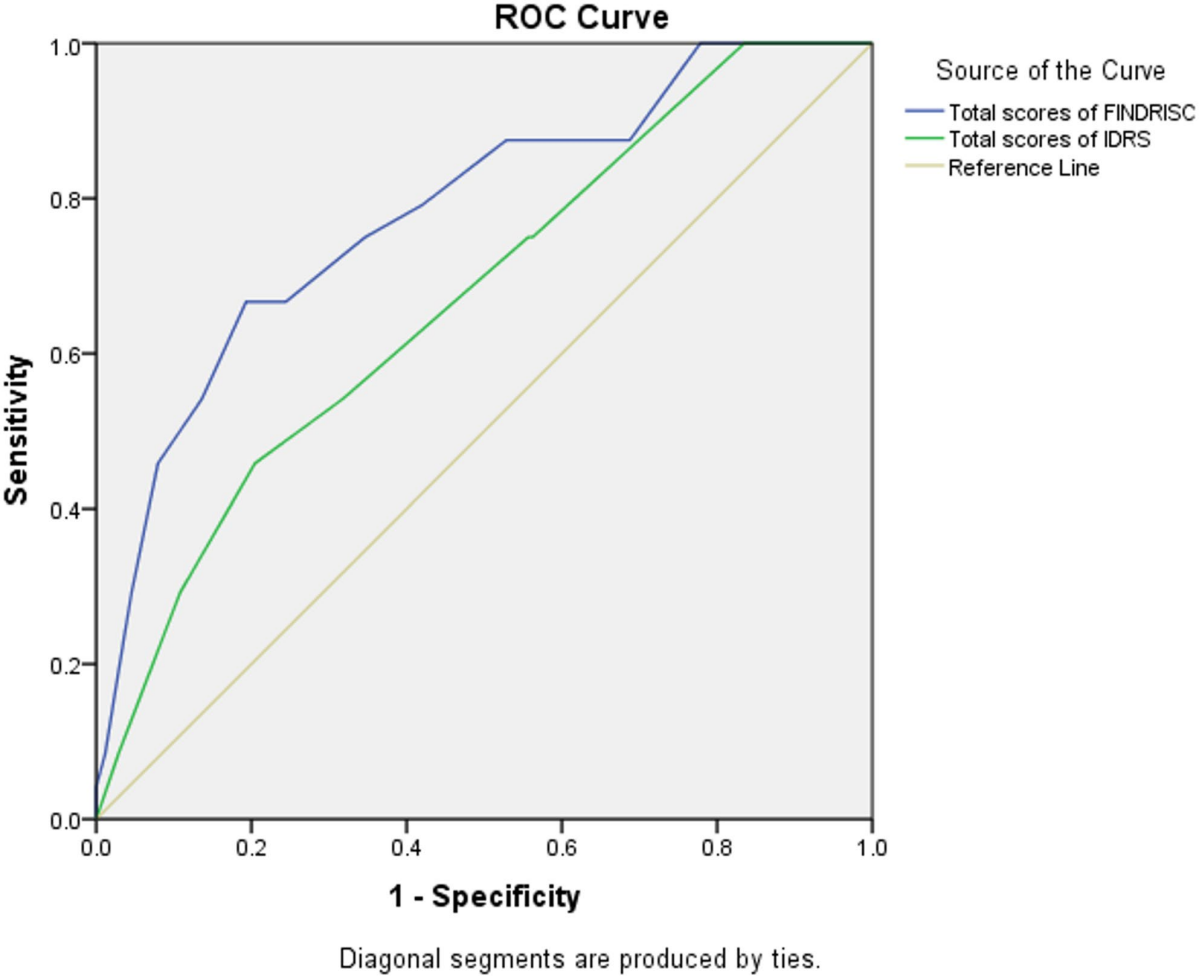
Comparison of area under the ROC curve (AUROC) for the FINDRISC score and IDRS score

We further analyzed the data to validate the IDRS score and FINDRISC against increased fasting blood sugar levels for detecting dysglycemia (FBS: 100–125 or ≥ 126 mg/dl). When assessing the diagnostic accuracy of the FINDRISC score and IDRS score for stratifying the risk of dysglycemia (Table 5), the AUC for FINDRISC was 0.78 (95% CI: 0. 68–0.88), with an optimal cut-off of ≥ 9.0, yielding a Youden Index of 0.44, a sensitivity of 67.0%, and a specificity of 81.0%. In contrast, the AUC for the IDRS was 0.67 (95% CI: 0.56–0.78), with a cut-off of ≥ 45; this yields a Youden Index of 0.25, a sensitivity of 46.0% and a specificity of 80.0%, which provided the highest balanced accuracy for this population. There was a significant difference between the AUCs of both scores (*P* < 0.001). As shown in Fig. 3, the AUC in our study was significantly larger for FINDRISC than for IDRS (0.78 vs. 0.67), indicating superior diagnostic accuracy for the FINDRISC in this particular Yemeni population for identifying dysglycemia.

Compared to the IDRS, the FINDRISC showed higher sensitivity (67.0% vs.46.0%), while both models revealed comparable specificity (81.0% and 80.0%, respectively). The FINDRISC also exhibited a higher positive likelihood ratio than IDRS (3.5 vs. 2.3). This indicates that subjects classified as high‑risk by the Finnish were 3.5 times more likely to have dyglycemia than those not at risk, showing a stronger clinical utility than the IDRS (0.21 vs. 0.11).

**Table 5:** Comparative diagnostic performance of FINDRISC and IDRS scores for detecting dysglycemia

Screening characteristic	FINDRISC score	IDRS score
AUC (95%CI)	0.78	0.67
Youden index	0.44	0.25
Optimal cut-off	9.0	45.0
Sensitivity	67.0	46.0
Specificity	81.0	80.0
Positive likelihood ratio	3.50	2.30
Negative likelihood ratio	0.41	0.68
Diagnostic accuracy	79.0	75.5
Clinical Utility Index (CUI+)	0.21	0.11
Clinical Utility Index (CUI-)	0.80	0.73

**Fig. 4:**
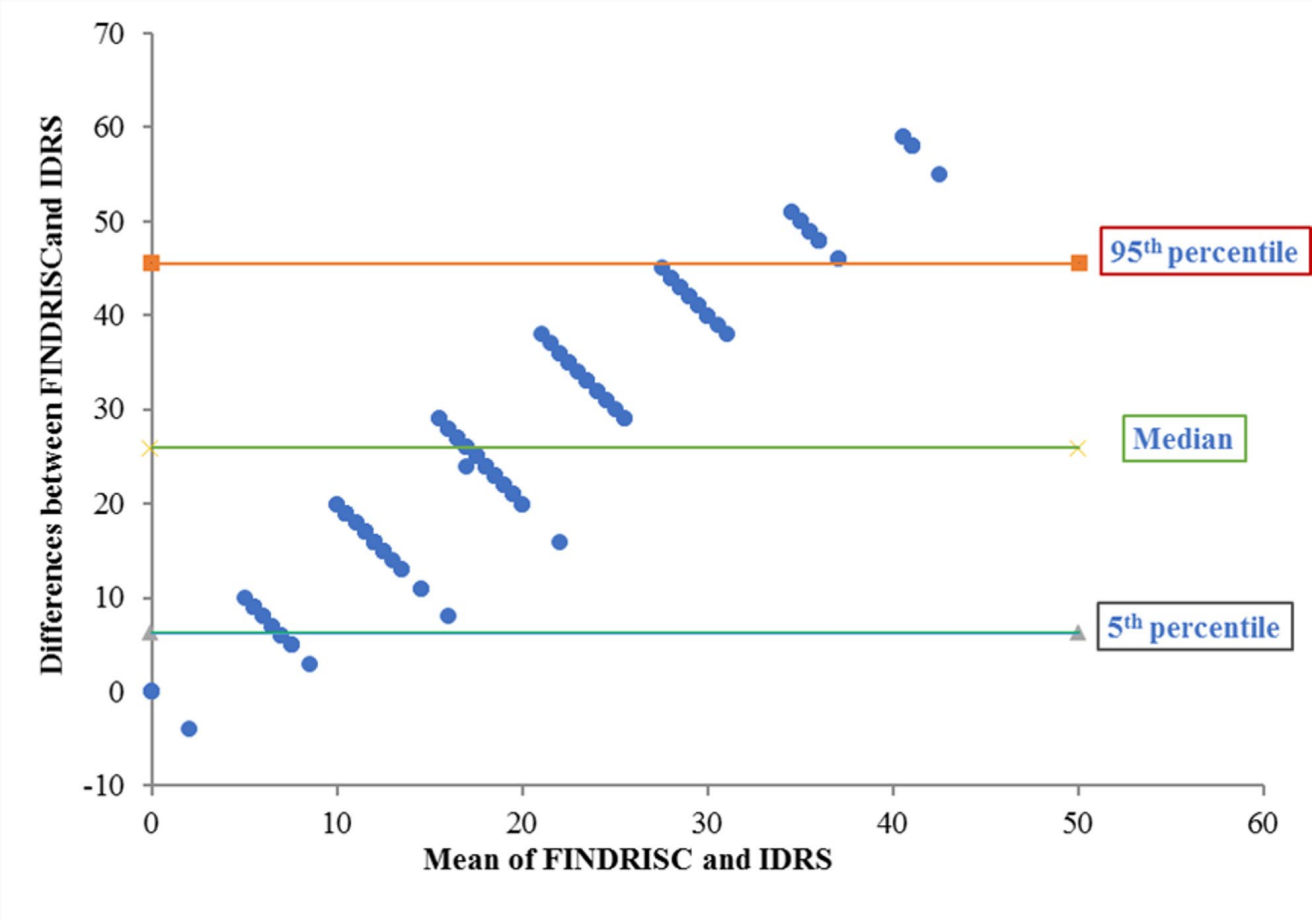
The Bland-Altman plot for assessing the agreement between Finnish Diabetes Risk Score (FINDRISC) and Indian Diabetes Risk Score (IDRS)

When comparing accuracy (79% for FINDRISC vs. 75% for IDRS) and clinical utility indices (CUI) of the two models, both were found to be very poor for case finding. However, the clinical utility of FINDRISC was “very good” for ruling out dysglycemia (CUI- : 0.80 vs. 0.73). Additionally, we measured the level of agreement between FINDRISC and IDRS scores by using a Bland-Altman (B-A) plot (Fig. 4). The plot shows that differences were increased as the mean value increased, indicating no perfect agreement. The majority of values fall within the 5th–95th percentiles, highlighting a critical proportionality bias: the two tools demonstrate adequate agreement at lower risk levels but diverge significantly as the risk score increases.

We also compared the applicability of the two risk score models in detecting dysglycemia (undiagnosed T2DM [UT2DM] and prediabetes [PDM]) among the study subjects using standard clinical practice cut-off values (Table 6). According to the FINDRISC score, 14(7.0%) subjects were categorized as high risk (FINDRISC score ≥ 12); among these, 7 had dysglycemia. Notably, Only one of these 7 dysglycemic subjects had a FINDRISC score ≥ 15. It is worth mentioning that lowering the FINDRISC cut-off value to ≥ 9, resulted in the highest detection of dysglycemia (8.5%) compared to the ≥ 12 threshold (3.5%). In contrast, we found that there was no significant difference in detecting dysglycemia when using IDRS cut-off values of ≥ 45 or ≥ 60 (*P* = 0.06). However, the IDRS cut-off value ≥ 45 was effective for screening PDM than the ≥ 60 threshold (Table 6).

**Table 6:** Comparison of different cut-offs for FINDRISC and IDRS scores for screening dysglycemia (*N* = 200)

	Non-diabetes (< 100 mg/dl) *n* = 176(%)	Prediabetes (100–125 mg/dl) *n* = 21(%)	Diabetes (≥ 126 mg/dl) *n* = 3(%)	*p*-value
**FINDRISC score**				
< 9	143(81.2)	7 (33.3)	0.0 (0.0)	< 0.001
≥ 9	33 (18.8)	14 (66.7)	3 (100.0)	
< 12	168 (95.5)	15 (71.4)	2 (66.7)	
≥ 12	8 (4.5)	6 (28.6)	1 (33.3)	
**IDRS score**				
< 45	140 (79.5)	11 (52.4)	2 (66.7)	0.06
≥ 45	36 (20.5)	10 (47.6)	1 (33.3)	
< 60	157(89.2)	15 (71.4)	2 (66.7)	
≥ 60	19 (10.8)	6 (28.6)	1 (33.3)	

*Results are expressed as expressed as frequency and percentage *n*(%). Chi-Square *p* < 0.05 considered as significant value

Summarily, at a cut-off point of FINDRISC ≥ 9, of the total participants, 17(8.5%) were identified with dysglycemia; of these, 14(7.0%) had prediabetes and 3 (1.5%) had diabetes. In contrast, at an IDRS cut-off point of ≥ 45, only 11(5.5%) participants were identified with dysglycemia, comprising of 10 (5.0%) with prediabetes and only1 (0.5%) with diabetes.

## Discussion

Diabetes is increasingly identified as an important cause of mortality and a critical public health issue worldwide [4, 28]. An important burden for diabetes, besides a health burden, is the financial burden to individuals and society. This is attributed to its high prevalence and the high cost of diabetic care per individual [28]. It has been reported that the early identification of high risk individuals using easy, noninvasive, cost effective, and reliable tools can help reduce or prevent the development of T2DM and its associated cardiometabolic complications through dietary and lifestyle interventions [29, 30]. For this purpose, several diabetes risk screening models have been developed to be easily and effectively used in clinical practice with minimum cost [31]. However, it is not ensured whether these risk score tools can be applied in local populations. Data suggest that some tools developed for a specific population do not always perform well in other populations [32]. Thus, the applicability of these models in a different population is limited and can be misleading. Therefore, various countries need to continue to explore diabetes assessment systems that are suitable and applicable for local populations [33].

In Yemen, despite the widespread use of diabetes risk scores, no previous study, to the best of our knowledge, has studied the discriminatory accuracy of different assessment scores for diabetes screening in a young Yemeni population. Hence, this study compares the screening effectiveness of two frequently used, less costly validated risk scores — the FINDRISC and IDRS among a young medical student population in Taiz city, Yemen. The main reasons for T2DM screening in this population group include their sedentary lifestyles, the increasing incidence of diabetes in younger populations, and limited studies conducted among them.

Based on the data of our study, the overall prevalence of dysglycemia was 12%. This finding is consistent with previous studies by Viitasalo et al. and Sapkota et al., who found a prevalence of 12.9% and 11.3%, respectively, among their study participants [34, 35]. However, a recent study carried out in Yemen found a lower prevalence of 5.7% [36]. In contrast to our finding, studies conducted in different countries among adult population. For example, studies by Al-Shudifat et al., Ali et al., D’Souza et al., and Nnamudi et al., which reported varying prevalence of 3.8%, 8.2%, 26.9% and 32.8% respectively [22, 23, 37, 38]. This disparity could be due to differences in the high number of participants at a high risk of diabetes, that is, older and obese, and along with the use of varying cutoff values for hyperglycemia to define PDM and T2DM, such as the inclusion of the oral glucose tolerance test alongside impaired fasting glucose.

A noteworthy finding in our study was the low prevalence of PDM (10.5%) and UT2DM (1.5%) among the participants. This finding is inconsistent with the results of a study by Farag et al., who reported a prevalence of 21.7% and 5%, respectively among their study participants [39]. Similarly, a study at the Lebanese University showed a prevalence of 22.9% for PDM and 7.6% for UT2DM [24]. Conversely, studies in different countries reported a lower prevalence of PDM among young adult students [40–42]. Nevertheless, increasing awareness of the alarming global prevalence of PDM is crucial for young subjects to implement preventive measures. Regarding undiagnosed T2DM, it was well demonstrated that about 1.5% of subjects were unaware of their disease. This inconsistency in PDM and UT2DM prevalence may arise from differences in the participant characteristics (age, sex, ethnicity), sampling technique, diagnostic criteria, lifestyle choices, unhealthy dietary habits, socioeconomic status, and genetic factors.

It is worth mentioning that there was a significant difference in the risk of dysglycemia between the sexes (*P* < 0.001). This could be explained by the higher frequency of overweight and obesity associated with sedentary lifestyles, limited physical activity and poor nutritional behaviors among young adult females in our region. This finding aligns with studies by Ahmad et al. and Evcimen et al., which also found a significantly higher diabetic risk among females [40, 41]. In contrast to our findings, a previous study done by Sezer et al., which found no statistically significant difference between females and males regarding their risk of developing T2DM [42]. This discrepancy may be partly due to differences in sample sizes, risk factors included in study tool, or variations in the populations involved.

The prevalence and risk factors of diabetes in a population determine the applicability of a risk score. In this study, the prevalence of T2DM increased with increasing FINDRISC and IDRS scores. While Several cross-sectional studies have tested these tools, the discriminatory performance of the IDRS versus the FINDRISC score has not previously been evaluated in detecting dysglycemia in a Yemeni population. Furthermore, because both risk scores include variables common to Asian populations [43] and have been validated in neighboring Arab populations, their use as suitable screening tools in the current study is well-justified [22–24].

Regarding the accuracy and the validation of FINDRISC and IDRS in our study, their performance is generally analyzed based on sensitivity, specificity, and ROC curves. For IDRS, the best cut‑off was ≥ 45 for identifying dysglycemia, with a sensitivity of 46.0%, a specificity of 80.0%, and a Youden index of 0.25 (Table 5). This finding is in agreement with a study from central India that showed that the optimal cut-off for IDRS was ≥ 40 (sensitivity 60.4%, specificity70.7%, and Youden index 0.31) [44]. Conversely, Pawar et al. found a sensitivity of 78.95% and specificity of 56.14% at an optimal cutoff of > 60 [45]. This disparity might be attributed to ethnic diversity, differences in the populations studied, or variations in the categorization of physical exercise across various studies. Notably, the low sensitivity of the IDRS in our study, coupled with high specificity, suggests that while the test accurately identifies subjects who do not have diabetes (high true negatives), it may be missing a significant number of subjects who do have diabetes (high false negatives). This suggests the IDRS is more effective at ruling out dysglycemia with a low level of accuracy (at Youden index of 0.25) than identifying it.

On the other hand, the FINDRISC had shown an optimal cut-off of ≥ 9 for identifying dysglycemia, with a sensitivity of 67.0%, a specificity of 81.0%, and a Youden index of 0.44 (Table 5). This demonstrates that at a cut-off of ≥ 9, the FINDRISC correctly identifies approximately 67% of subjects with UT2DM and 81% of those without the disease, reflecting a moderate level of accuracy (at Youden index of 0.44). This finding aligns with previous studies that also identified a FINDRISC cut-off of ≥ 9 as the optimal threshold for detecting UT2DM [10, 32]. For examples, in two retrospective cohorts, Bhowmik et al. found sensitivities of 62.4% and 75.7%, and specificities of 67.4% and 61.6%, respectively, at a risk score of ≥ 9 [46]. Variations in the study populations could be the possible reason for the differences in sensitivity and specificity values observed at the same cut-off point across various studies [46].

Overall, the results of our study demonstrated that the optimal cut-off value for the FINDRISC was at ≥ 9, with a significantly larger area under the ROC curve (AUC: 0.78; 95% CI: 0.68–0.88) compared to the IDRS (AUC: 0.67; 95% CI: 0.56–0.78).While these results indicate that FINDRISC has acceptable discriminatory accuracy, its performance is considered modest; this suggests it is best utilized as an initial screening tool to detect individuals at high risk of having dysglycemia rather than a standalone diagnostic tool. The superior performance of FINDRISC over IDRS in this young population may be attributed to its inclusion of a broader range of metabolic risk factors [45].

The suboptimal performance of the IDRS likely arises from its original calibration for an older Indian demographic. The tool heavily weights age and specific physical activity categories that may not effectively capture the metabolic risk profile of young Yemeni adults. Because the IDRS thresholds are optimized for populations with higher age-related risk, the tool lacks the sensitivity required for this young population. Consequently, its ability to categorize risk is limited compared to the FINDRISC, which comprises a more diverse array of metabolic and lifestyle variables that maintain diagnostic relevance in younger populations. Agreement between the two tools was further evaluated using a Bland-Altman plot. Although the majority of observations fell within the 5th − 95th percentiles, a notable proportionality bias was observed. Specifically, the two tools demonstrated adequate agreement at lower risk levels but diverged significantly as the mean risk score increased. This bias suggests that the tools are not interchangeable, particularly for high-risk individuals.

Finally, the FINDRISC proved more effective in identifying subjects with dysglycemia in the high-risk category, as evidenced by the higher identified prevalence of PDM and UT2DM (7% and 1.5%, respectively) compared to the IDRS model (5% and 0.5%). As noted by Schmid et al., the proportion of subjects classified as ‘high-risk’ can vary significantly depending on the specific assessment tool utilized [47]. Given its moderate diagnostic accuracy, FINDRISC remains a pragmatic first-step approach.to identify subjects at high risk of dysglycemia, particularly in in resource-limited settings, such as in Yemen. Those with high risk scores could then to be subjected to more expensive or definitive tests to confirm the dysglycemia.

This study has several limitations especially in terms of potential bias and imprecision that should be considered when interpreting its findings. First, the study was conducted in a single University, which may raise questions regarding the generalizability of its findings to the broader young adult Yemeni population, which has diverse socioeconomic, and educational backgrounds. Furthermore, the study population in our study may not be representative of general population due to distinct characteristics, including higher health literacy, unique dietary patterns, and elevated stress levels, all of which may independently influence metabolic risk and diabetes prevalence. However, it is rather challenging to conduct such studies at the community level due to various logistical and ethical constraints. Furthermore, unlike other published studies in the country, this study provides valuable insights by prospectively comparing the accuracy performance FINDRISC and IDRS, thereby contributing to the existing literature in a country where such comparisons are lacking. Second, the small sample size may limit the statistical power to detect significant differences in certain characteristics or risk factors within specific subgroups. Additionally, low prevalence of dysglycemic cases (12%) restricts the ability to detect subtle differences between diagnostic methods within the dysglycemic subpopulation. Nevertheless, as a preliminary study, it still serves to reveal the performance and important differences between FINDRISC and IDRS as screening tools in the country’s young population. Moreover, its findings can serve as a guide for the design and implementation of large-scale studies that comprehensively investigate the diagnostic accuracy of FINDRISC and other diabetic risk scores for identifying individuals at risk of dysglycemia in Yemeni population. This gives a scientific answer to the question of which tool would be most appropriate to use in this situation. Third, the study employed the fasting blood glucose (FBS) test using spectrophotometer (BTS330) as the primary comparator, although the Oral Glucose Tolerance Test (OGTT) is often considered the preferred diagnostic method. Fourth, the use of self-reported key components for both scores (physical activity, family history, fruit/vegetable consumption) is prone to recall or reporting and social desirability bias. However, this study has several strengths. First, to this date, it is the first study comparing the accuracy performance of the FINDRISC versus IDRS among a medical student population in the Yemen. Second, this study comprised apparently healthy young adults, effectively excluding the chronic comorbidities and age-related complications typically prevalent in older populations.

## Conclusion

In conclusion, FINDRISC demonstrated acceptable diagnostic utility as an initial screening tool for identifying dysglycemia in this youngYemeni population. For resource-limited environments like the Yemeni health settings, the FINDRISC is preferred. While standalone sensitivity and AUC were modest (AUC = 0.78; Sensitivity = 67.0%), it significantly outperformed the IDRS in the study population. However, its moderate sensitivity suggests further laboratory investigations should be performed in individuals with a FINDRISC score (≥ 9) to confirm the diagnosis and ensure early detection of dysglycemia.

## Data Availability

Data are available from the corresponding author upon reasonable request.

## Abbreviations

AOR: Adjusted odds ratio
AUC: Area Under Curve
B-A plot: Bland–Altman Plot
BMI: Body Mass Index
CI: Confidence interval
CUIs: Clinical Utility Indices
FBS: Fasting blood sugar
FINDRISC: Finnish Diabetes Risk Score
IDF: International Diabetes Federation
IDRS: Indian Diabetes Risk Score
PDM: Prediabetes
ROC: Receiver Operating Characteristic Curve
SD: Standard deviation
SPSS: Statistical Package for the Social Sciences
T2DM: Type 2 Diabetes Mellitus
UT2DM: Undiagnosed Type 2 Diabetes Mellitus
WC: Waist Circumference
WHO: World Health Organization
